# Supplementary material for: Plumeriapropionics A–E, Carboxyl-Substituted Phenylpropionic Acid Derivatives with Anti-Inflammatory Activity from Plumeria rubra L
Source: Molecules. 2023 Dec 24;29(1):115. doi: 10.3390/molecules29010115 (PMC10779508; doi:10.3390/molecules29010115)
Supplement: Supplementary file 1 [file molecules-29-00115-s001.zip › molecules-2769988-supplementary.pdf]

Supplementary Material

List of Supplementary Material

Figure S1. ^{1}H NMR spectrum of plumeriapropionic A (**1**)

Figure S2. ^{13}C NMR spectrum of plumeriapropionic A (**1**)

Figure S3. 135DEPT spectrum of plumeriapropionic A (**1**)

Figure S4. HSQC spectrum of plumeriapropionic A (**1**)

Figure S5. HMBC spectrum of plumeriapropionic A (**1**)

Figure S6. ^{1}H-^{1}H COSY spectrum of plumeriapropionic A (**1**)

Figure S7. HRESIMS of plumeriapropionic A (**1**)

Figure S8. ^{1}H NMR spectrum of plumeriapropionic B (**2**)

Figure S9. ^{13}C NMR spectrum of plumeriapropionic B (**2**)

Figure S10. 135DEPT spectrum of plumeriapropionic B (**2**)

Figure S11. HSQC spectrum of plumeriapropionic B (**2**)

Figure S12. HMBC spectrum of plumeriapropionic B (**2**)

Figure S13. ^{1}H-^{1}H COSY spectrum of plumeriapropionic B (**2**)

Figure S14. HRESIMS of plumeriapropionic B (**2**)

Figure S15. ^{1}H NMR spectrum of plumeriapropionic C (**3**)

Figure S16. ^{13}C NMR spectrum of plumeriapropionic C (**3**)

Figure S17. 135DEPT spectrum of plumeriapropionic C (**3**)

Figure S18. HSQC spectrum of plumeriapropionic C (**3**)

Figure S19. HMBC spectrum of plumeriapropionic C (**3**)

Figure S20. ^{1}H-^{1}H COSY spectrum of plumeriapropionic C (**3**)

Figure S21. HRESIMS of plumeriapropionic C (**3**)

Figure S22. ^{1}H NMR spectrum of plumeriapropionic D (**4**)

Figure S23. ^{13}C NMR spectrum of plumeriapropionic D (**4**)

Figure S24. 135DEPT spectrum of plumeriapropionic D (**4**)

Figure S25. HSQC spectrum of plumeriapropionic D (**4**)

Figure S26. HMBC spectrum of plumeriapropionic D (**4**)

Figure S27. ^{1}H-^{1}H COSY spectrum of plumeriapropionic D (**4**)

Figure S28. HRESIMS of plumeriapropionic D (**4**)

Figure S29. ^{1}H NMR spectrum of plumeriapropionic E (**5**)

Figure S30. ^{13}C NMR spectrum of plumeriapropionic E (**5**)

Figure S31. HSQC spectrum of plumeriapropionic E (**5**)

Figure S32. HMBC spectrum of plumeriapropionic E (**5**)

Figure S33. ^{1}H-^{1}H COSY spectrum of plumeriapropionic E (**5**)

Figure S34. HRESIMS of plumeriapropionic E (**5**)

Figure S35. ECD spectra of **1** and **6**.

Figure S36. ECD spectra of **2** and **6**.

Figure S37. ECD spectra of **3** and **6**.

Figure S38. ECD spectra of **4** and **6**.

Figure S39. ECD spectra of **5** and **6**.

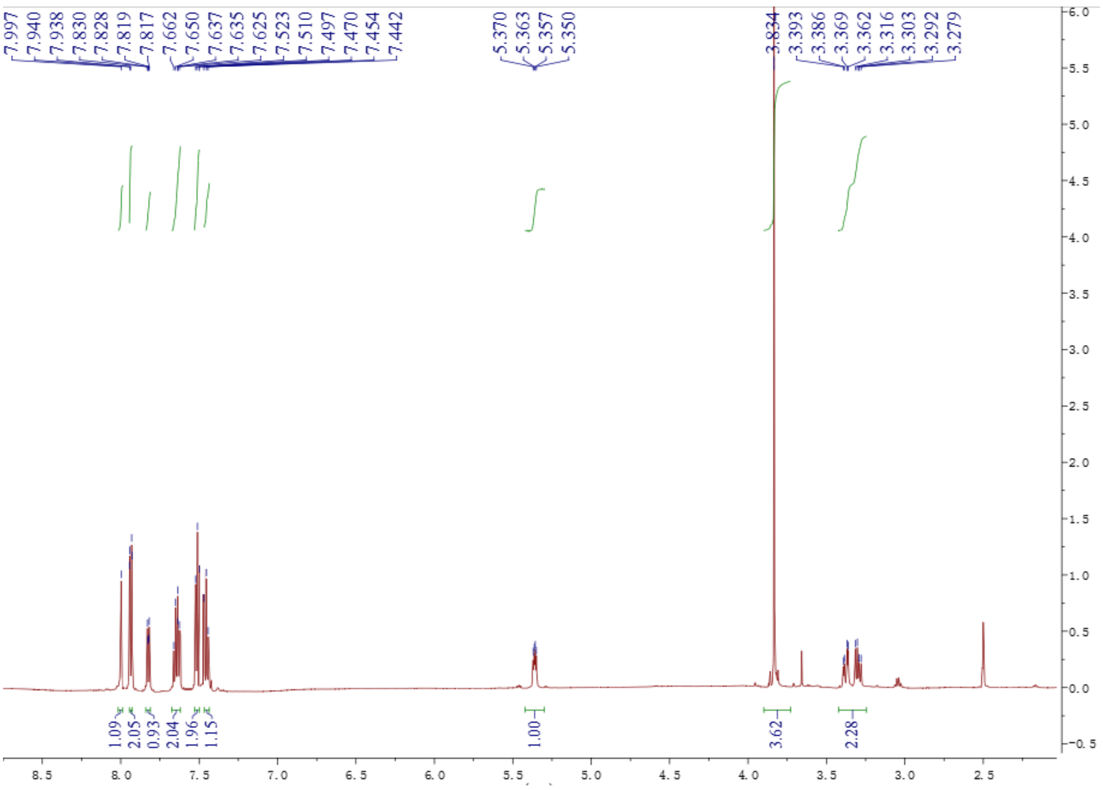

Figure S1. ^{1}H NMR spectrum of plumeriapropionic A (**1**)

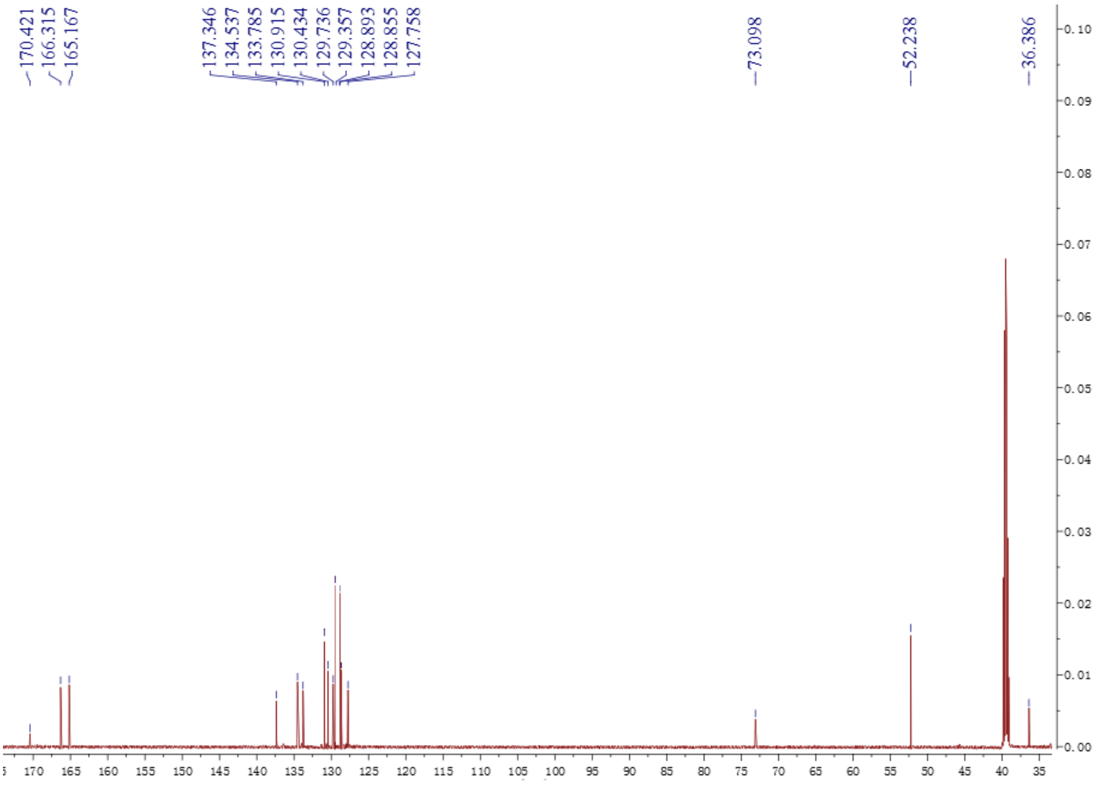

Figure S2. ^{13}C NMR spectrum of plumeriapropionic A (**1**)

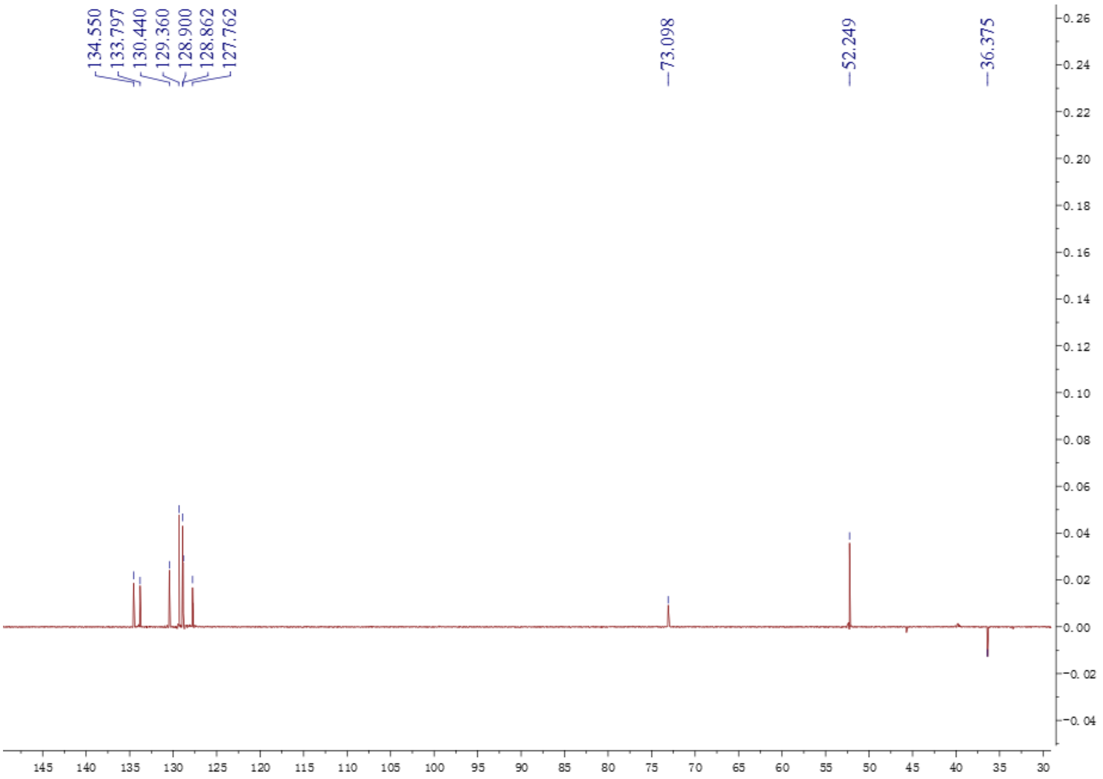

Figure S3. 135DEPT spectrum of plumeriapropionic A (**1**)

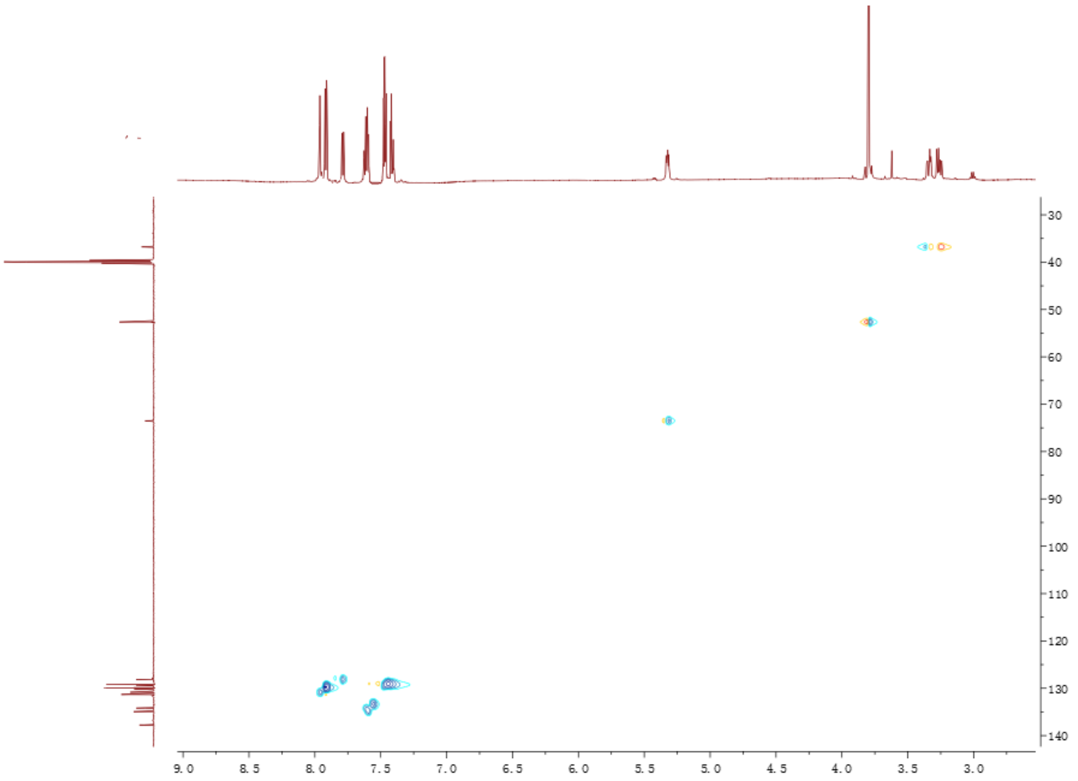

Figure S4. HSQC spectrum of plumeriapropionic A (**1**)

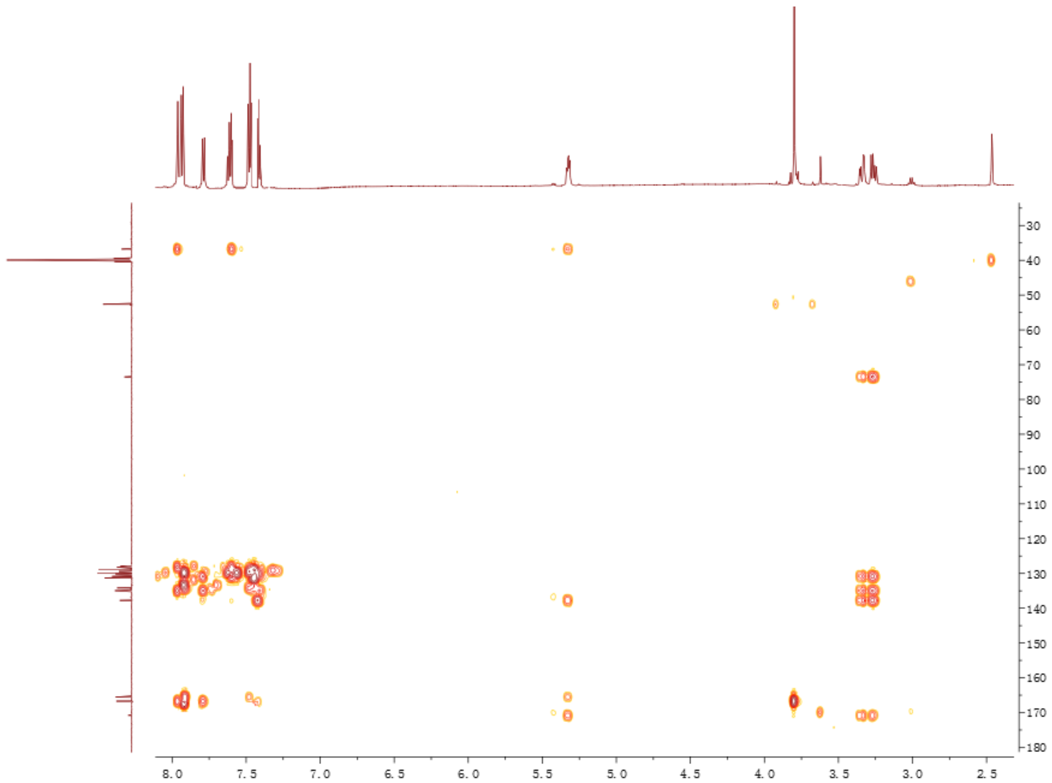

Figure S5. HMBC spectrum of plumeriapropionic A (**1**)

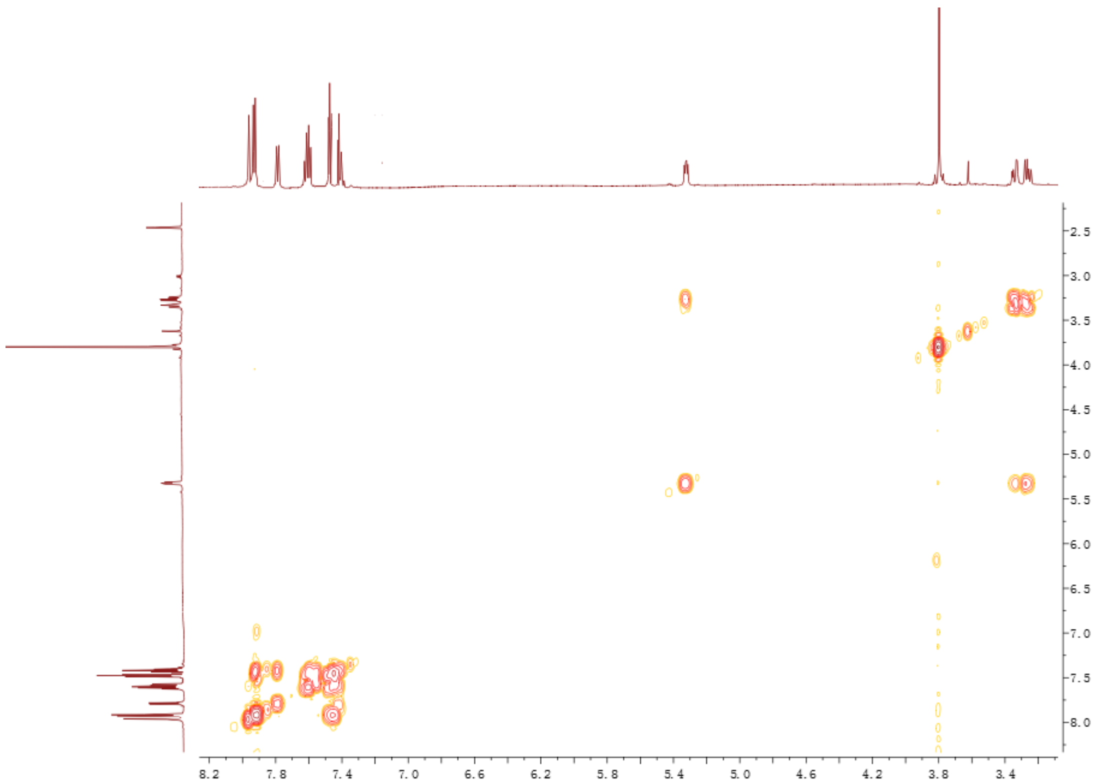

Figure S6. ^{1}H-^{1}H COSY spectrum of plumeriapropionic A (**1**)

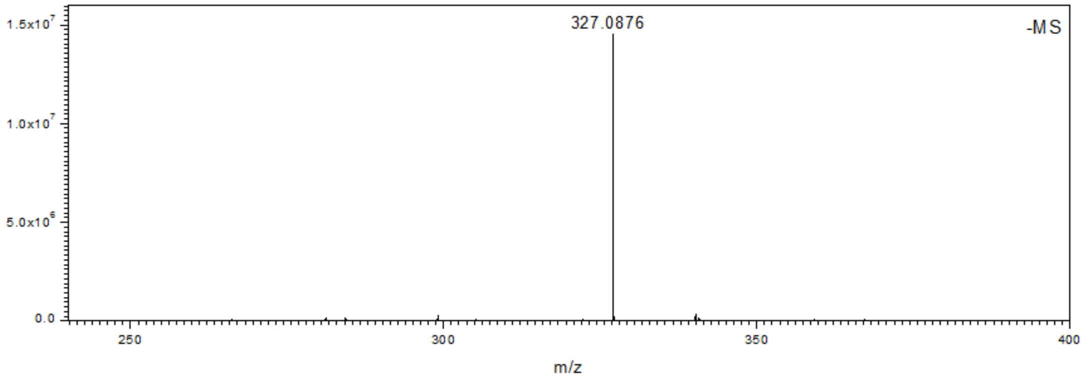

Figure S7. HRESIMS of plumeriapropionic A (**1**)

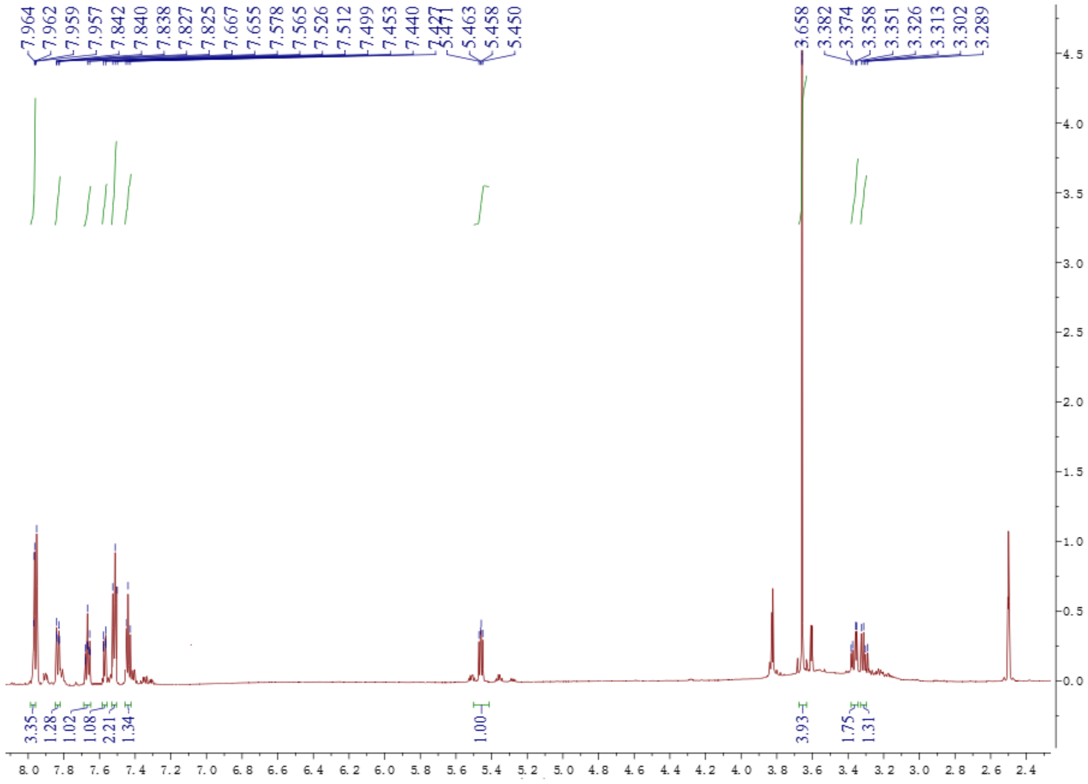

Figure S8. ^{1}H NMR spectrum of plumeriapropionic B (**2**)

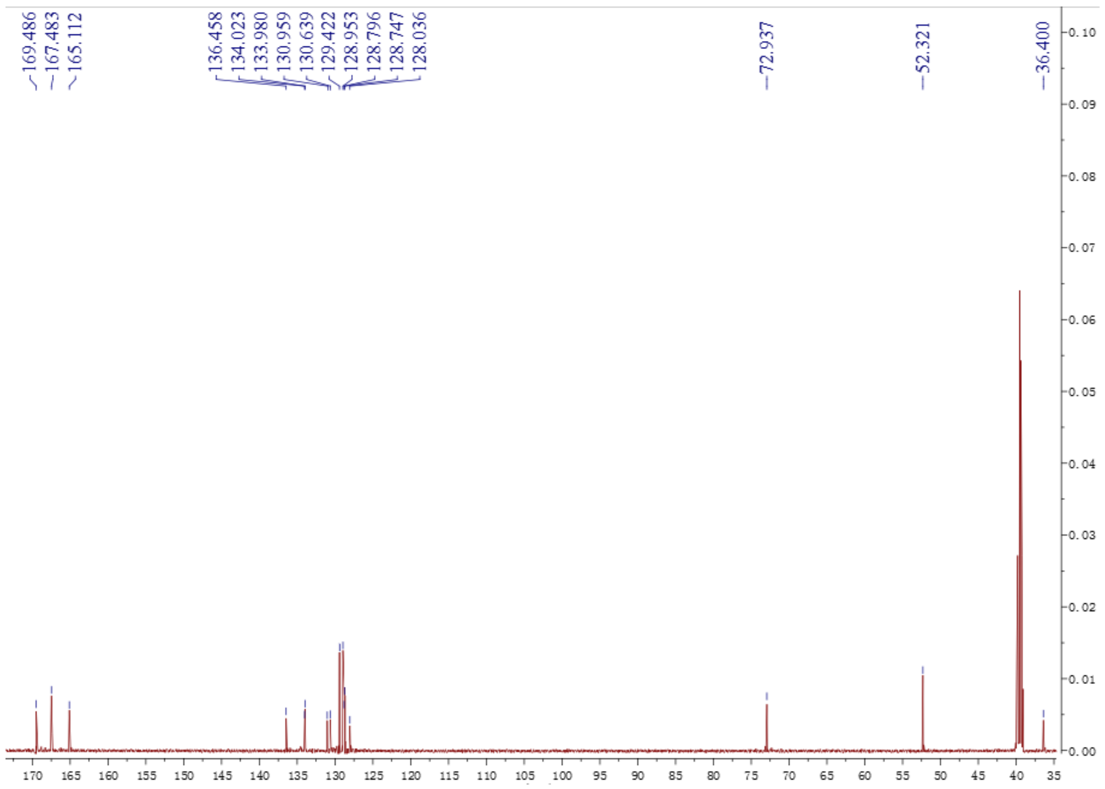

Figure S9. ^{13}C NMR spectrum of plumeriapropionic B (**2**)

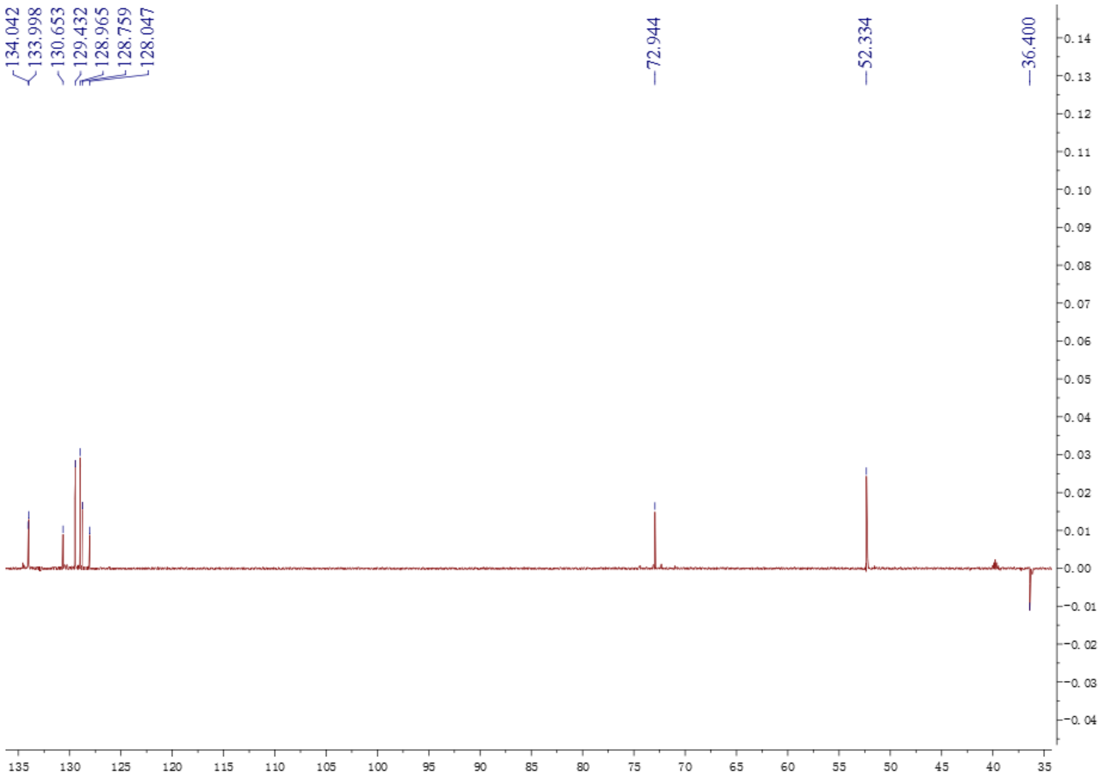

Figure S10. 135DEPT spectrum of plumeriapropionic B (**2**)

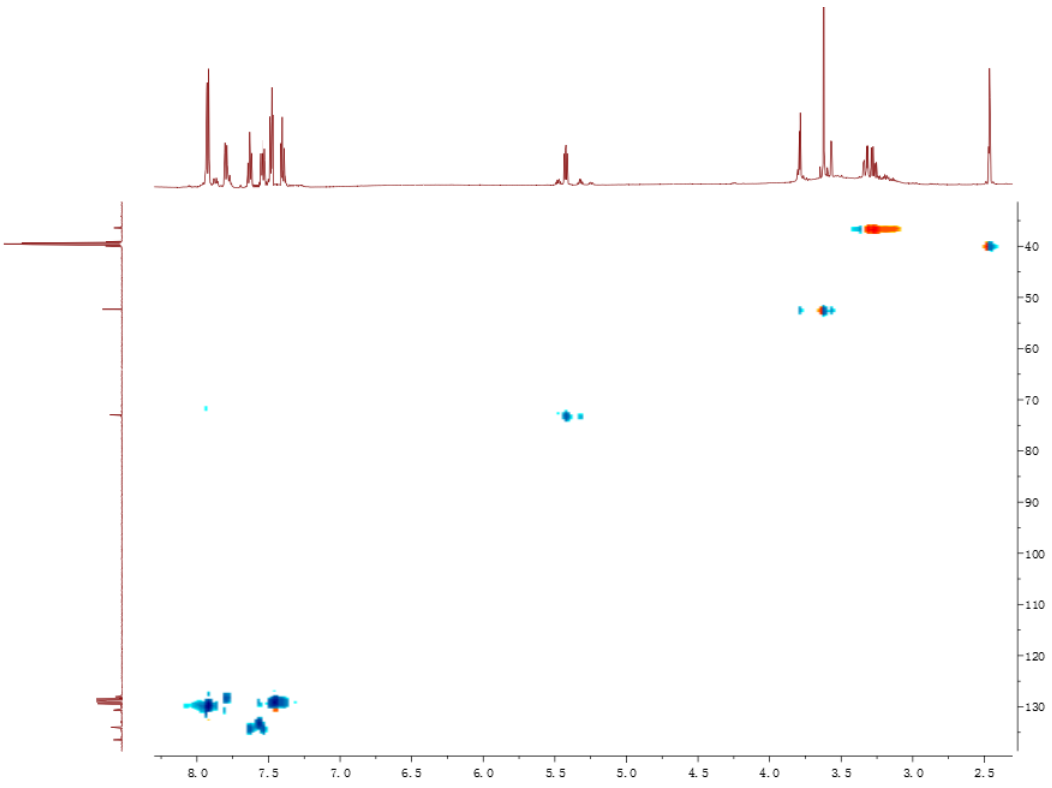

Figure S11. HSQC spectrum of plumeriapropionic B (**2**)

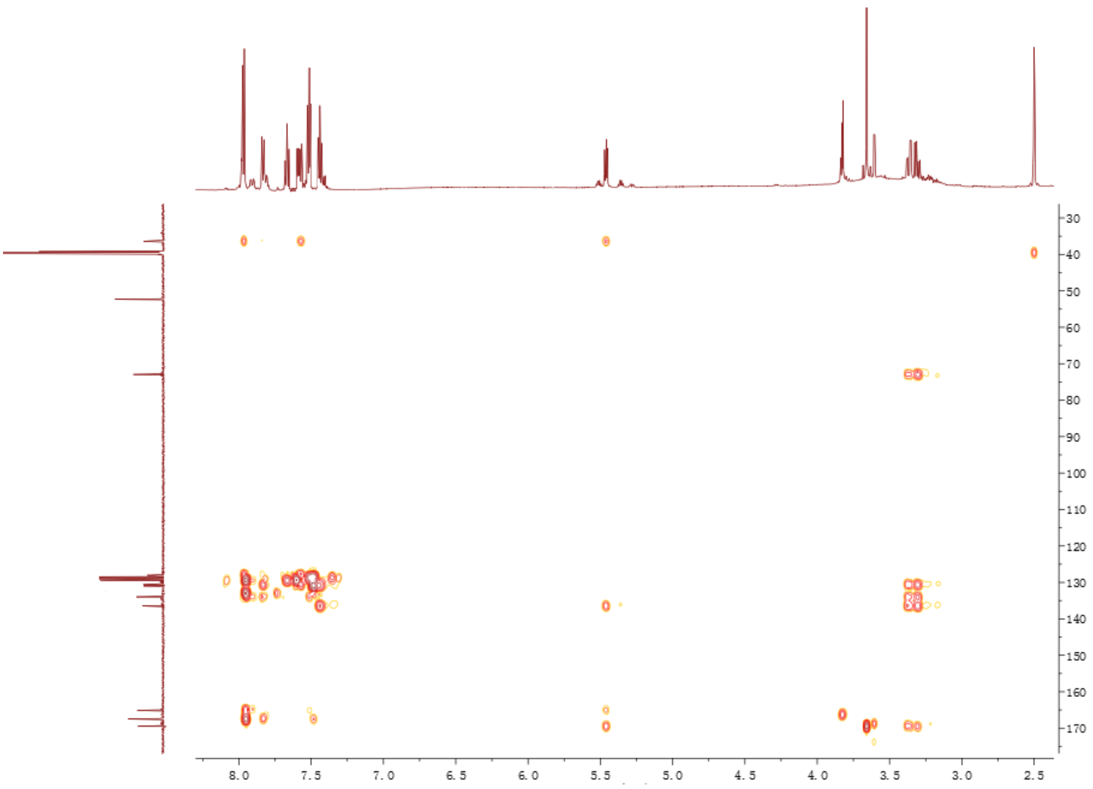

Figure S12. HMBC spectrum of plumeriapropionic B (**2**)

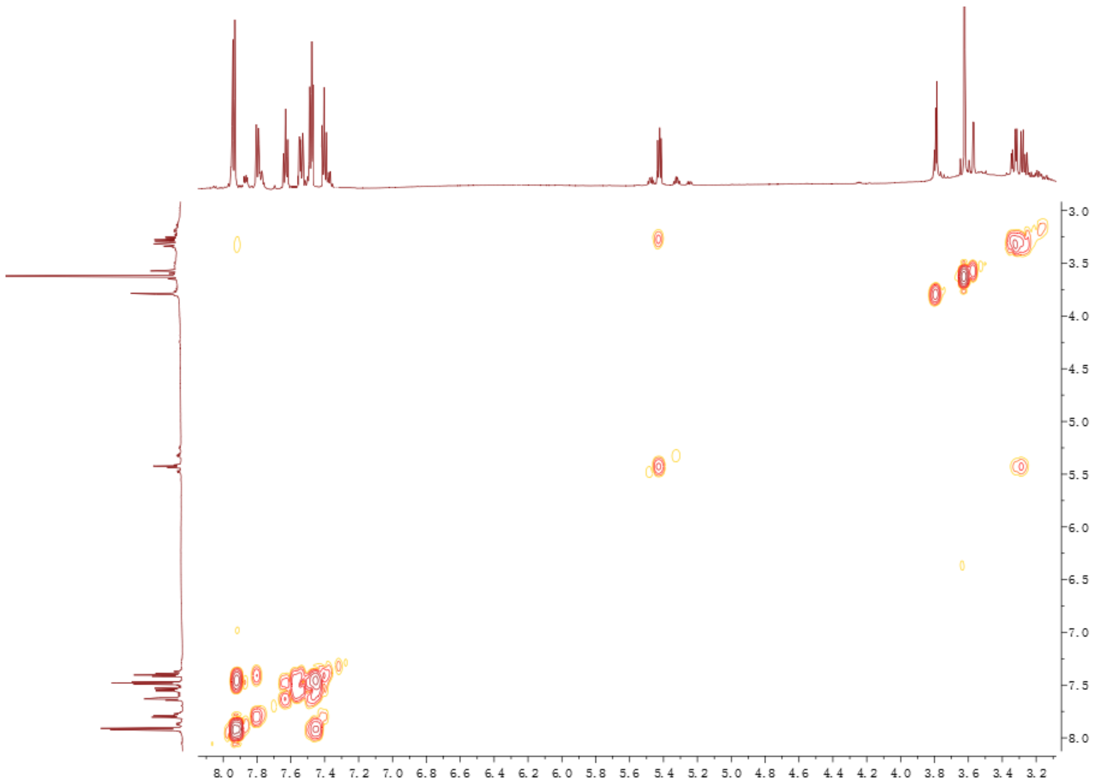

Figure S13. ^{1}H-^{1}H COSY spectrum of plumeriapropionic B (**2**)

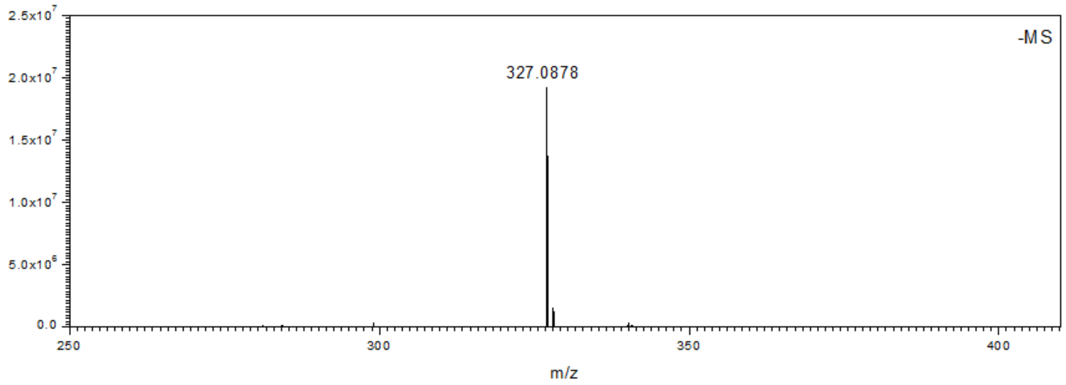

Figure S14. HRESIMS of plumeriapropionic B (**2**)

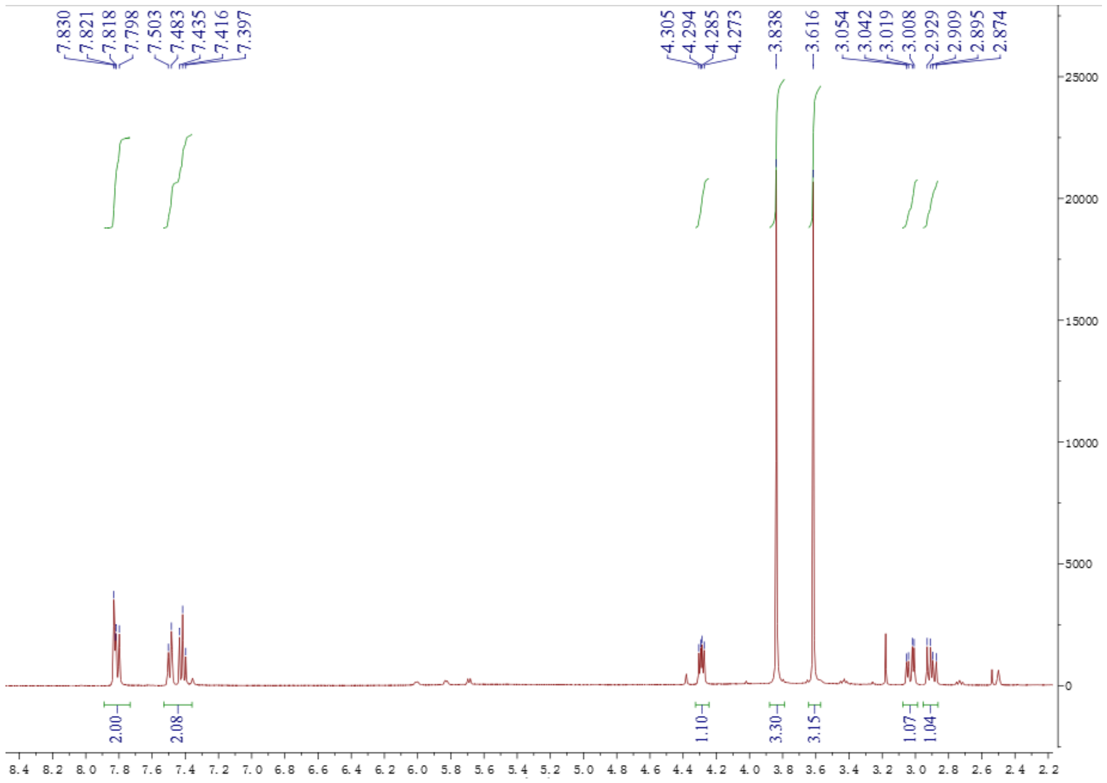

Figure S15. ^{1}H NMR spectrum of plumeriapropionic C (**3**)

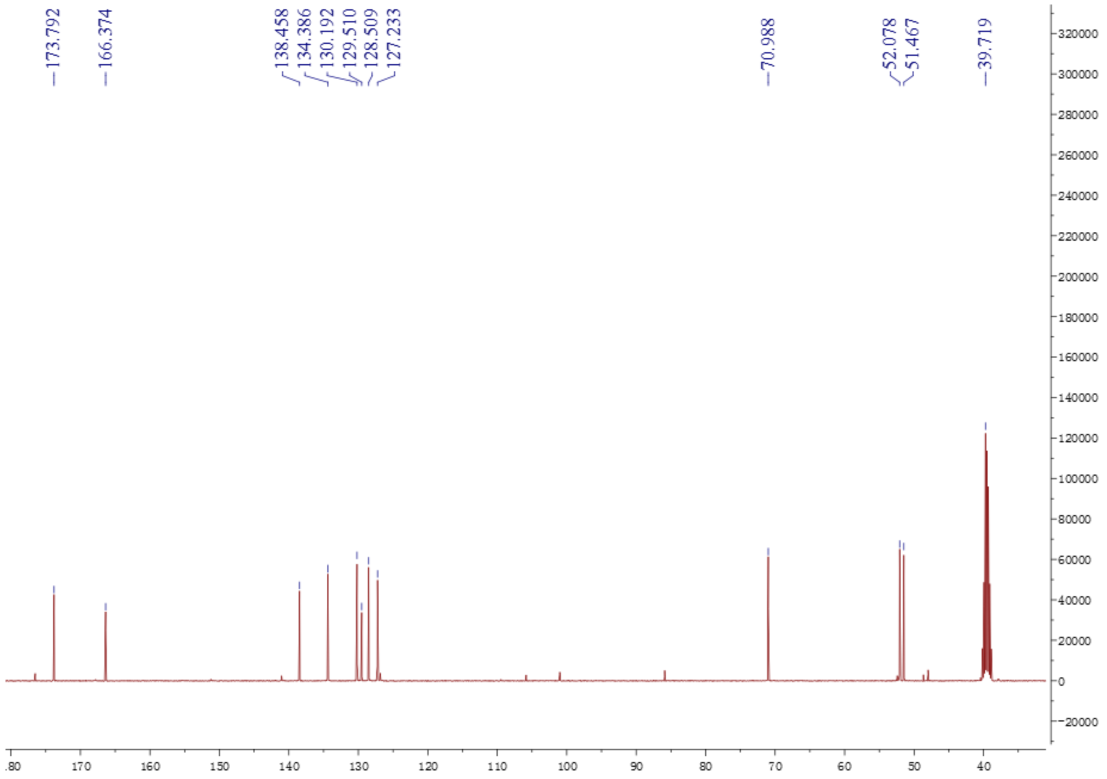

Figure S16. ^{13}C NMR spectrum of plumeriapropionic C (**3**)

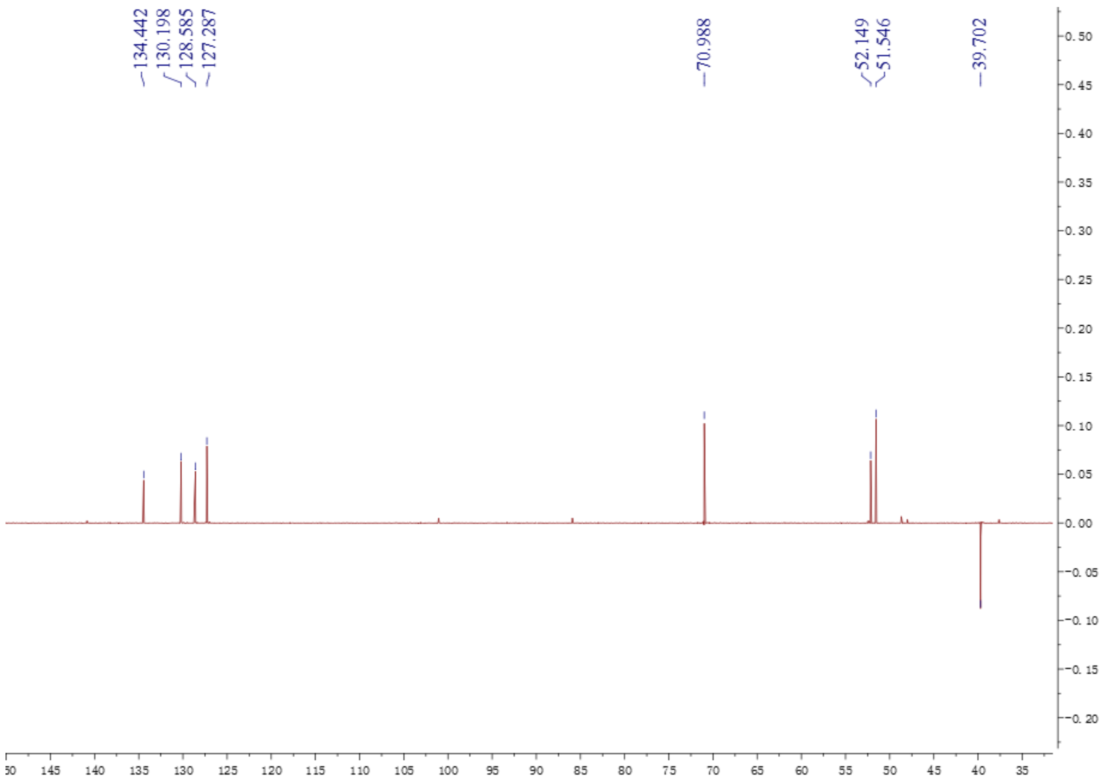

Figure S17. 135DEPT spectrum of plumeriapropionic C (**3**)

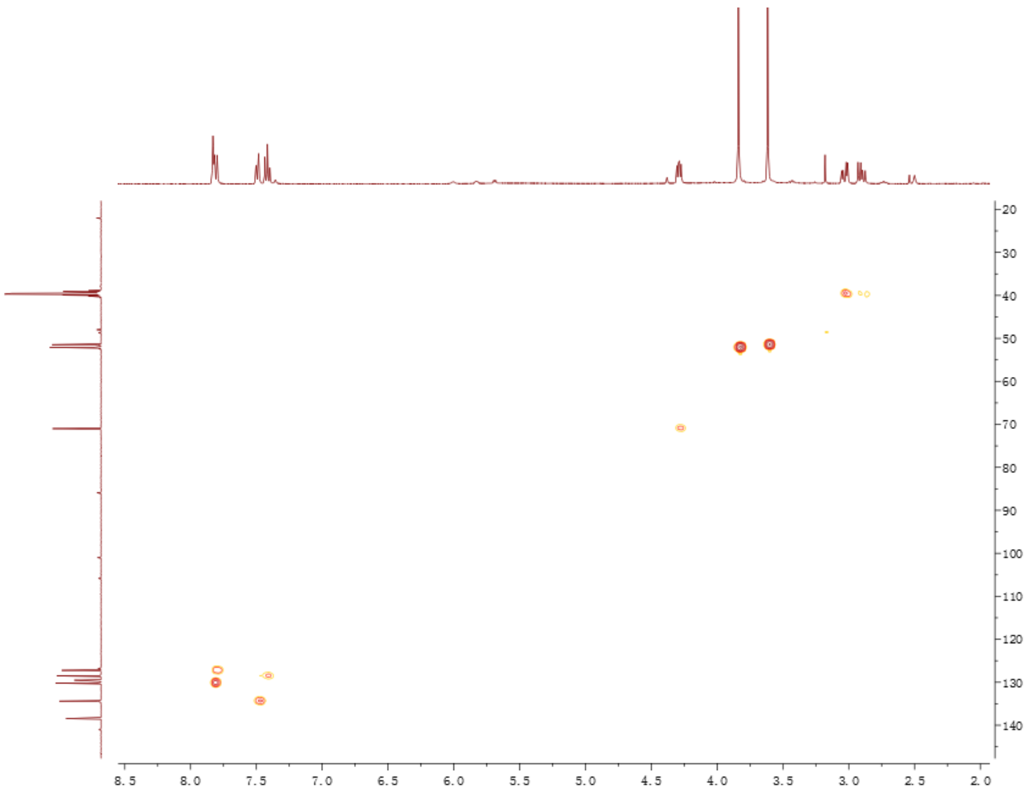

Figure S18. HSQC spectrum of plumeriapropionic C (**3**)

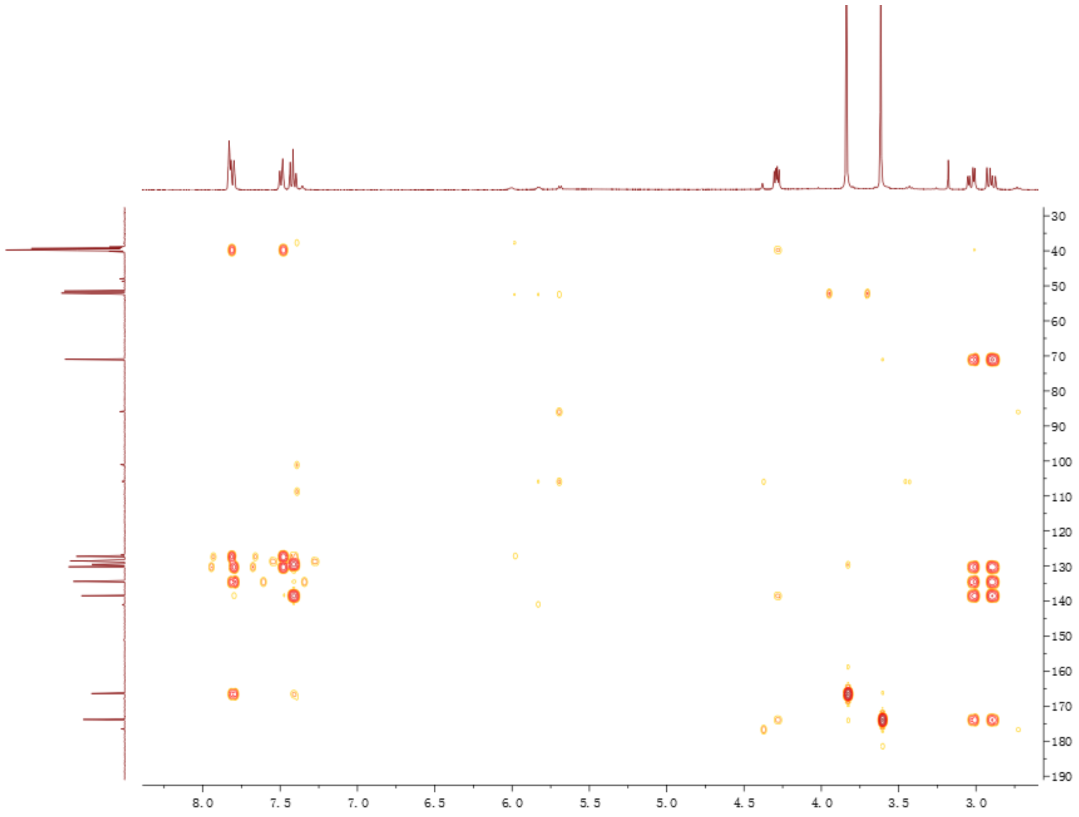

Figure S19. HMBC spectrum of plumeriapropionic C (**3**)

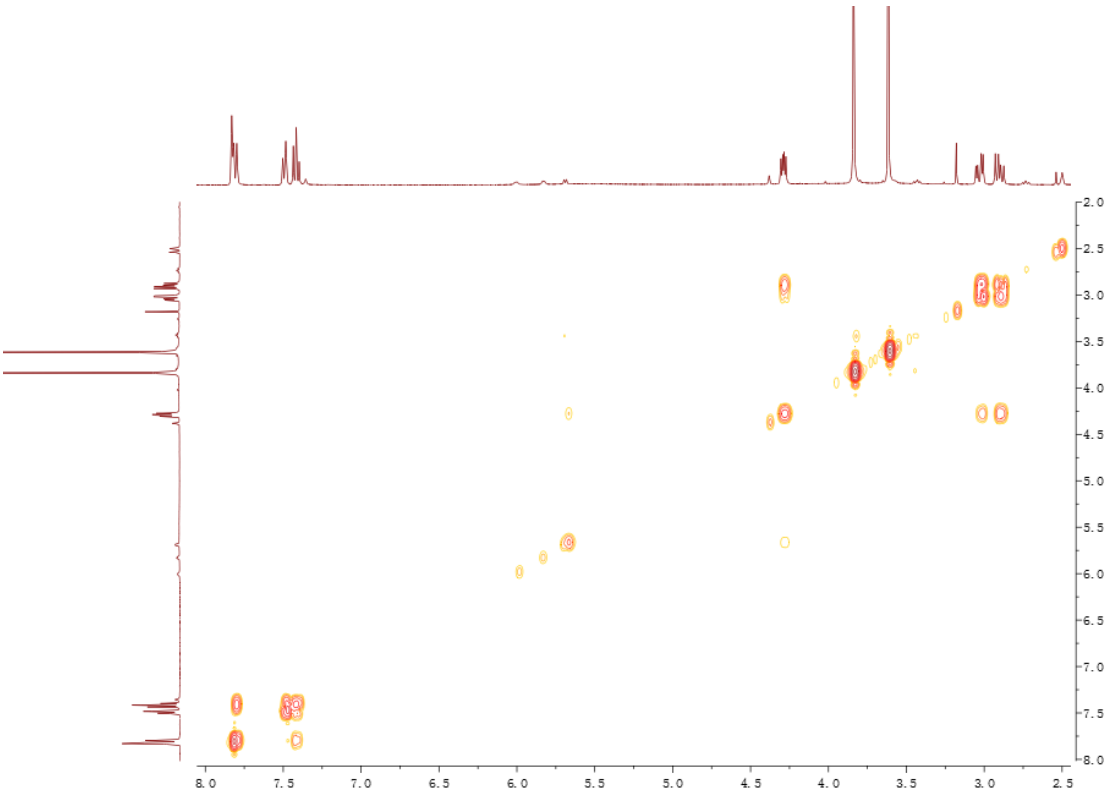

Figure S20. ^{1}H-^{1}H COSY spectrum of plumeriapropionic C (**3**)

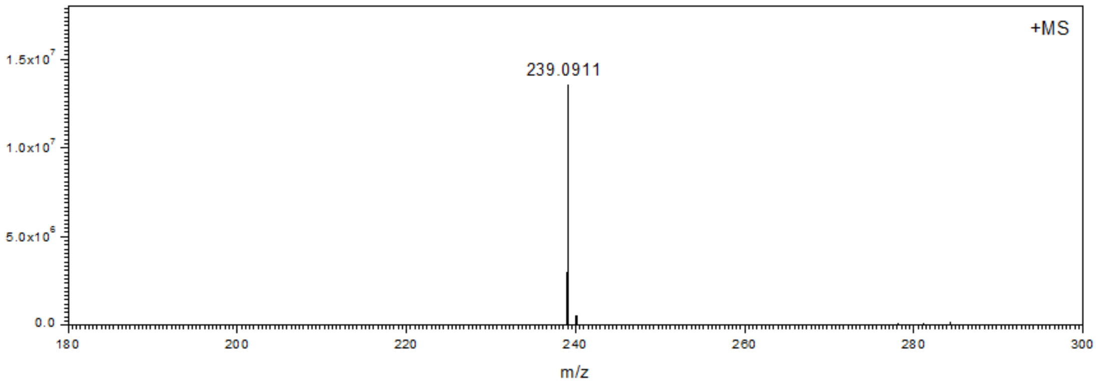

Figure S21. HRESIMS of plumeriapropionic C (**3**)

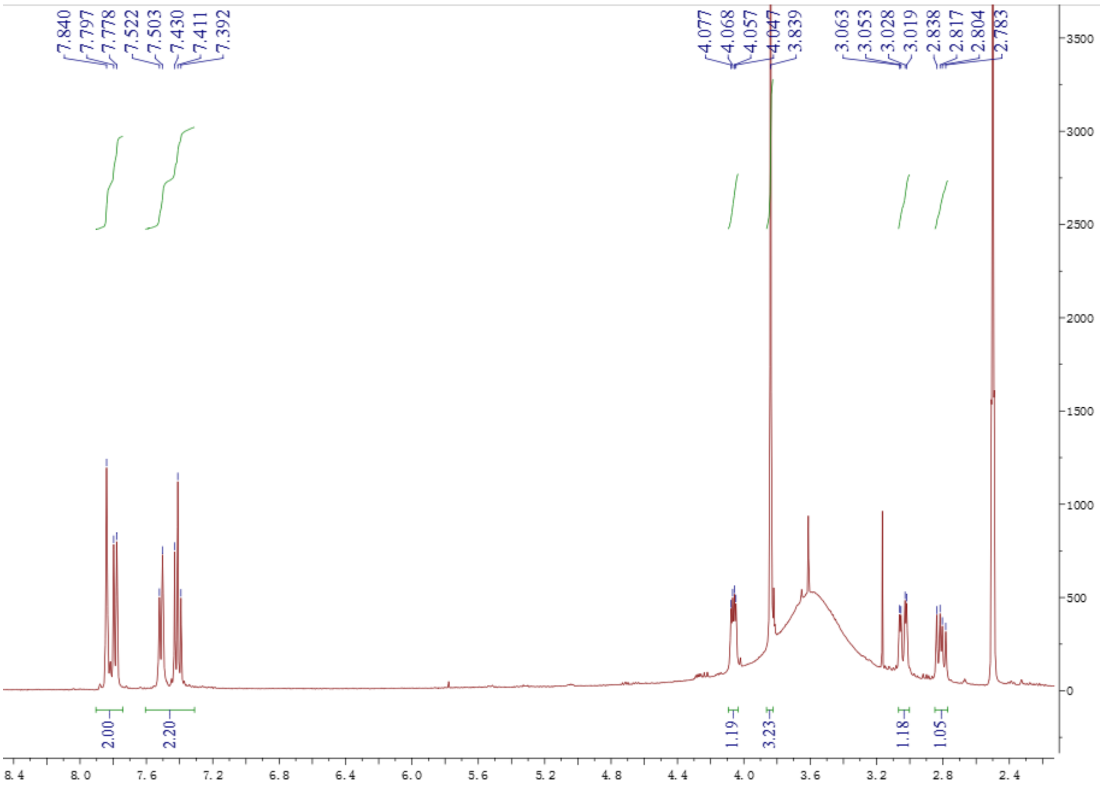

Figure S22. ^{1}H NMR spectrum of plumeriapropionic D (**4**)

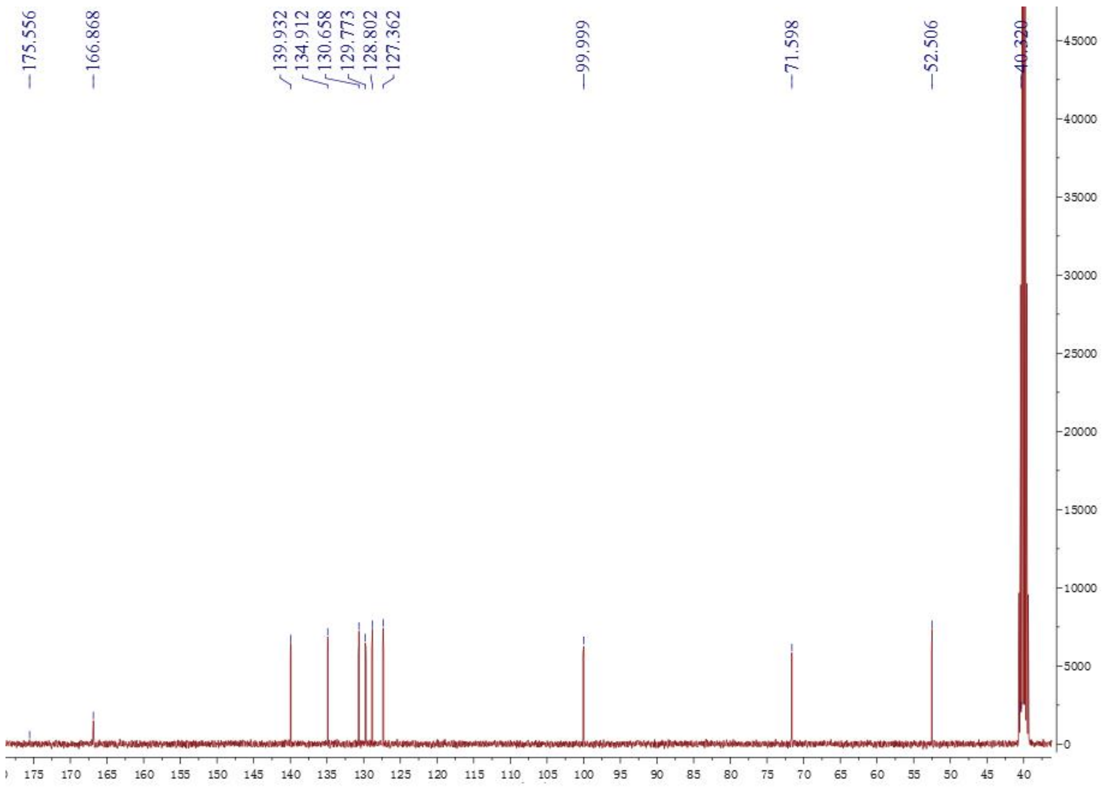

Figure S23. ^{13}C NMR spectrum of plumeriapropionic D (**4**)

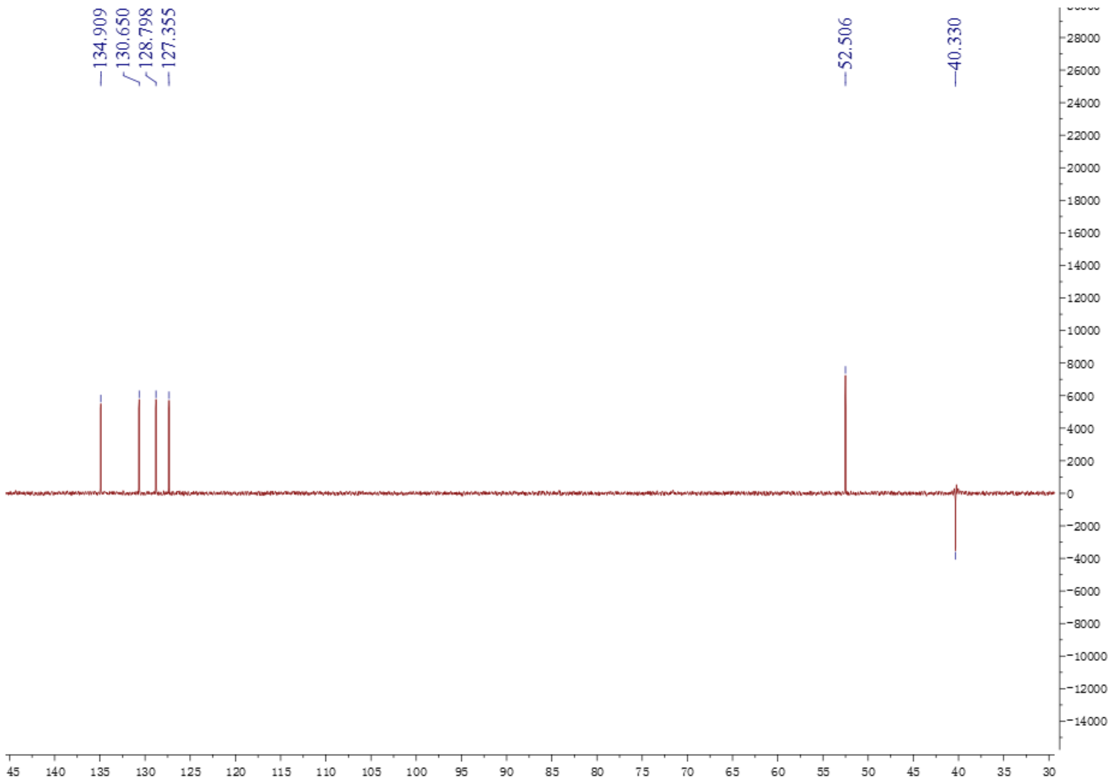

Figure S24. 135DEPT spectrum of plumeriapropionic D (**4**)

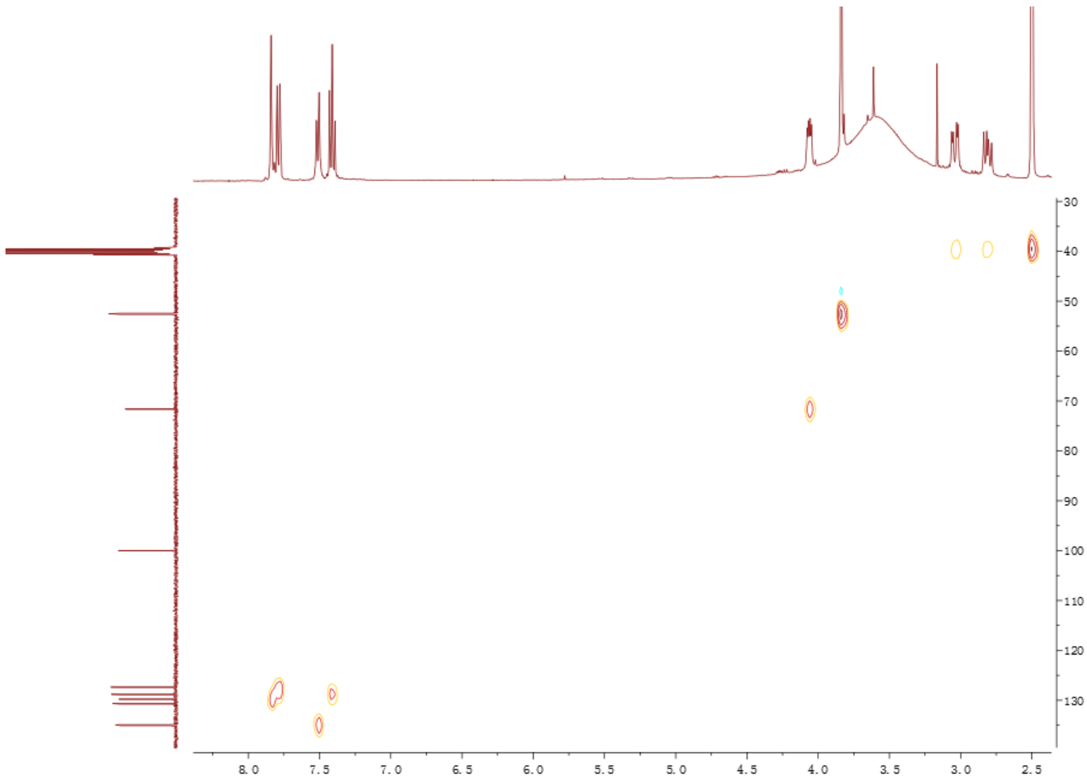

Figure S25. HSQC spectrum of plumeriapropionic D (**4**)

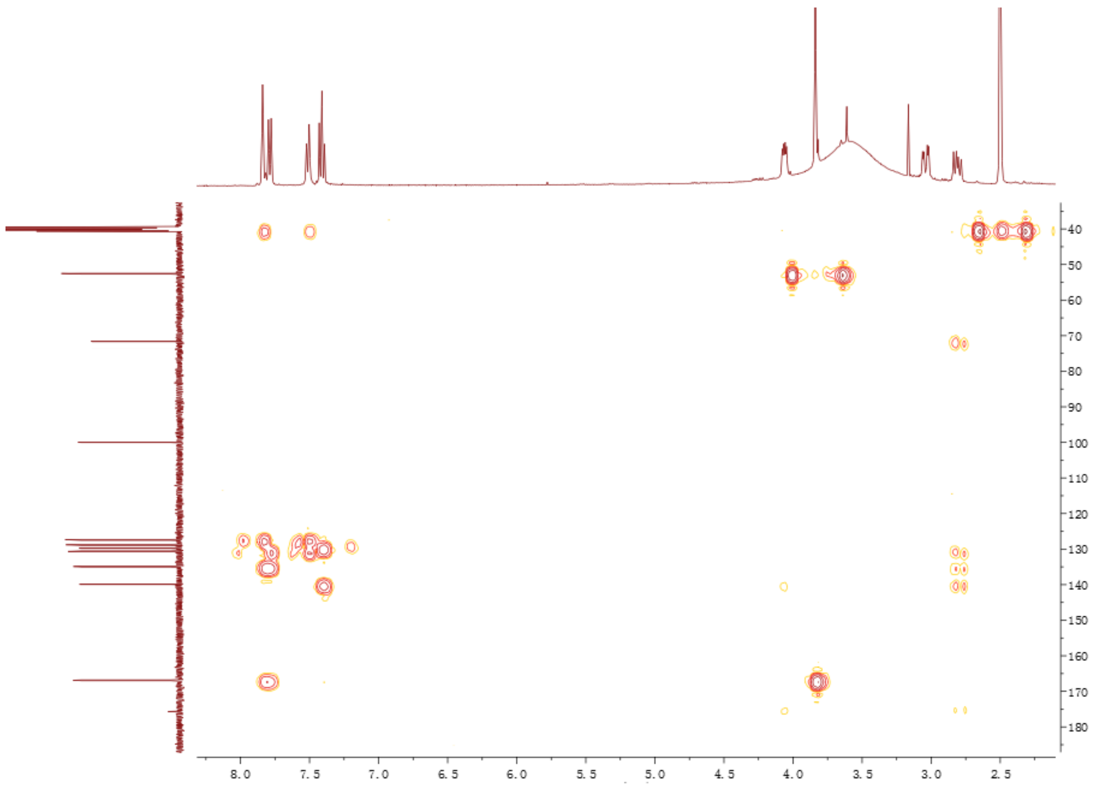

Figure S26. HMBC spectrum of plumeriapropionic D (**4**)

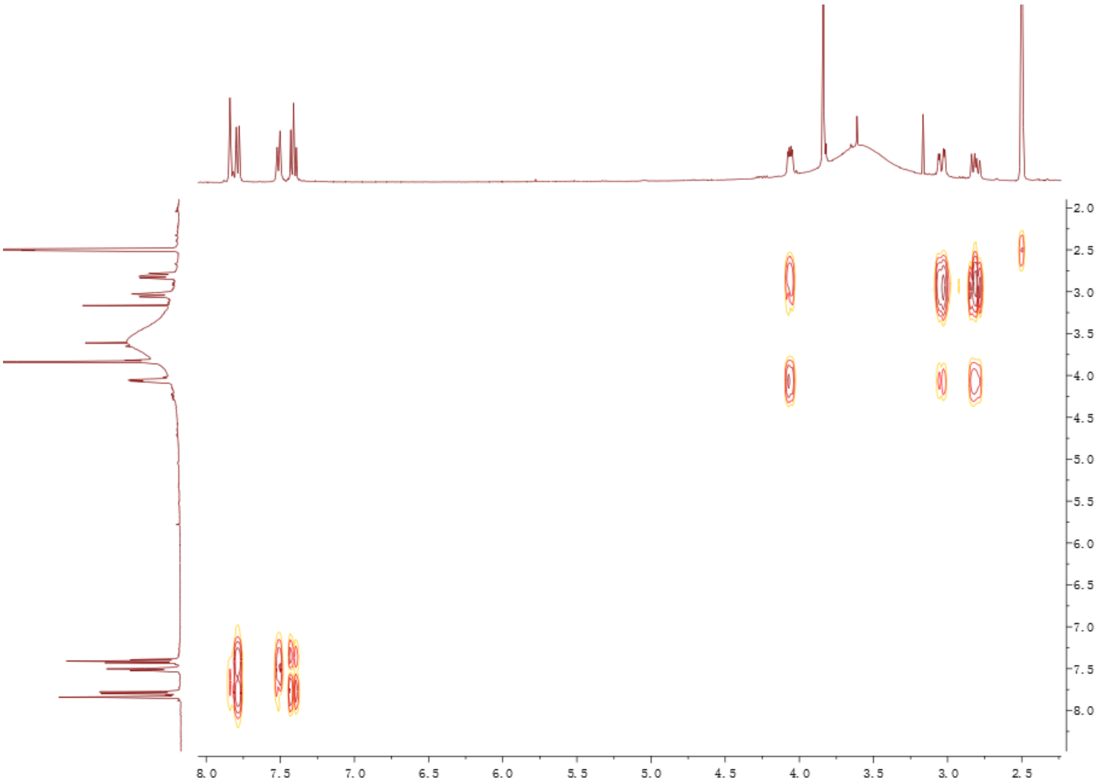

Figure S27. ^{1}H-^{1}H COSY spectrum of plumeriapropionic D (**4**)

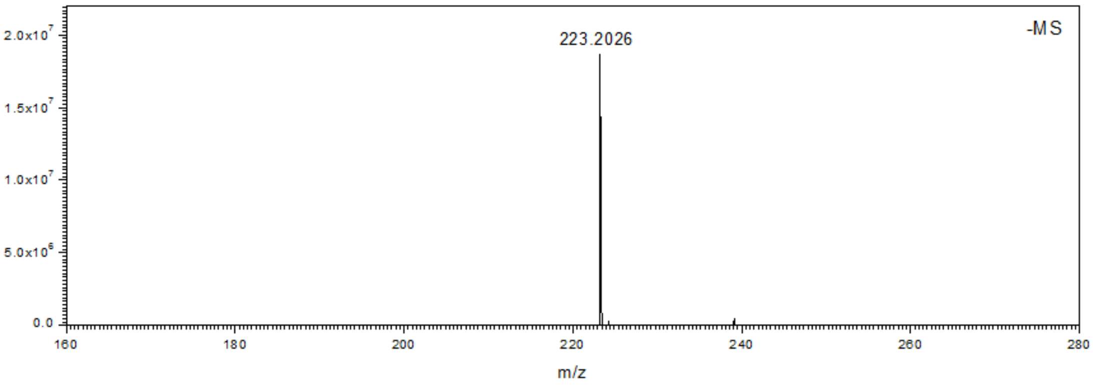

Figure S28. HRESIMS of plumeriapropionic D (**4**)

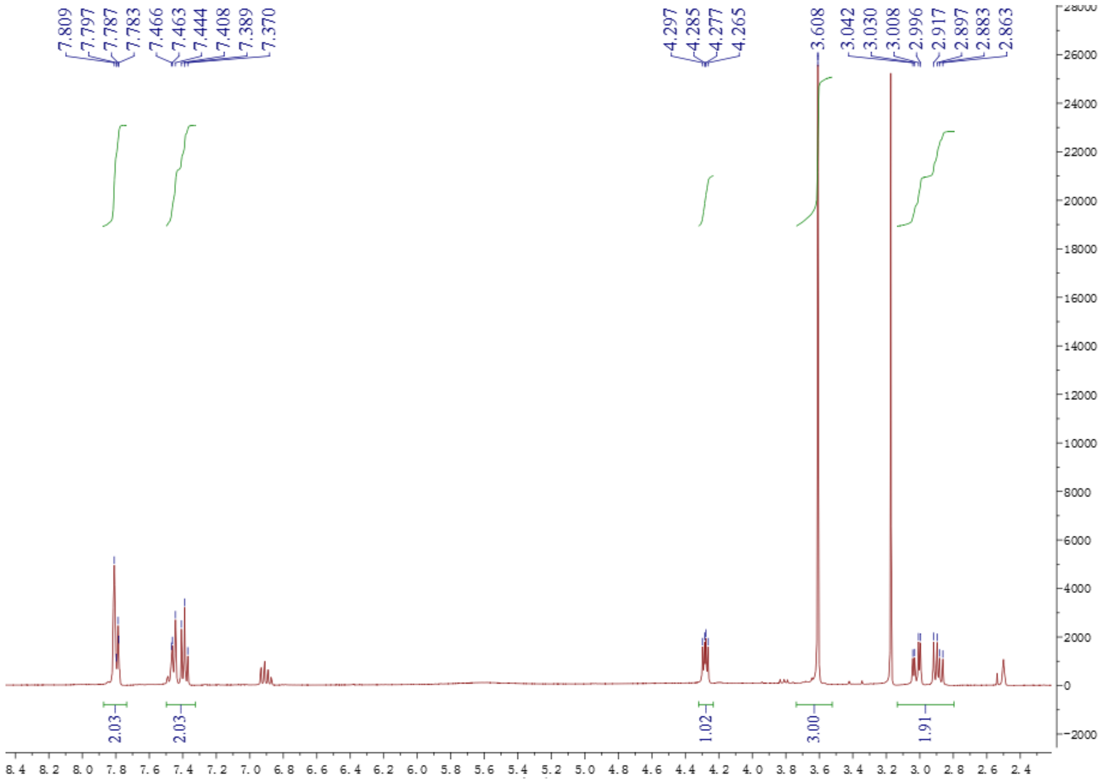

Figure S29. ^{1}H NMR spectrum of plumeriapropionic E (**5**)

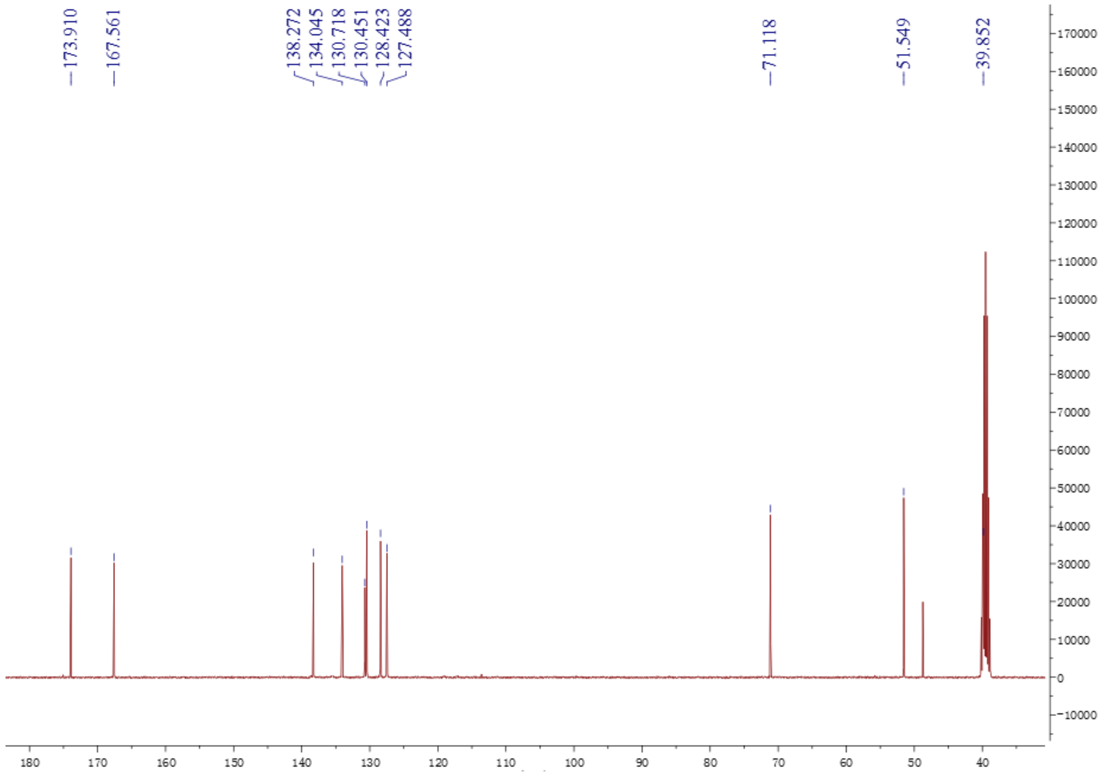

Figure S30. ^{13}C NMR spectrum of plumeriapropionic E (**5**)

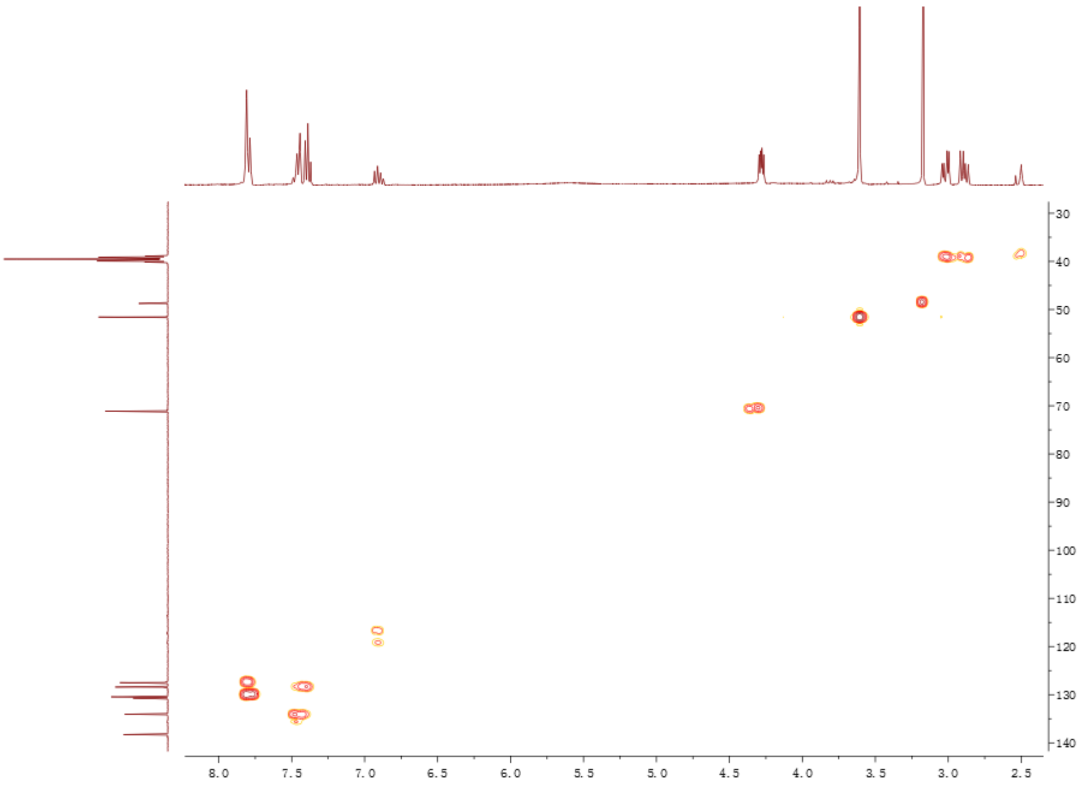

Figure S31. HSQC spectrum of plumeriapropionic E (**5**)

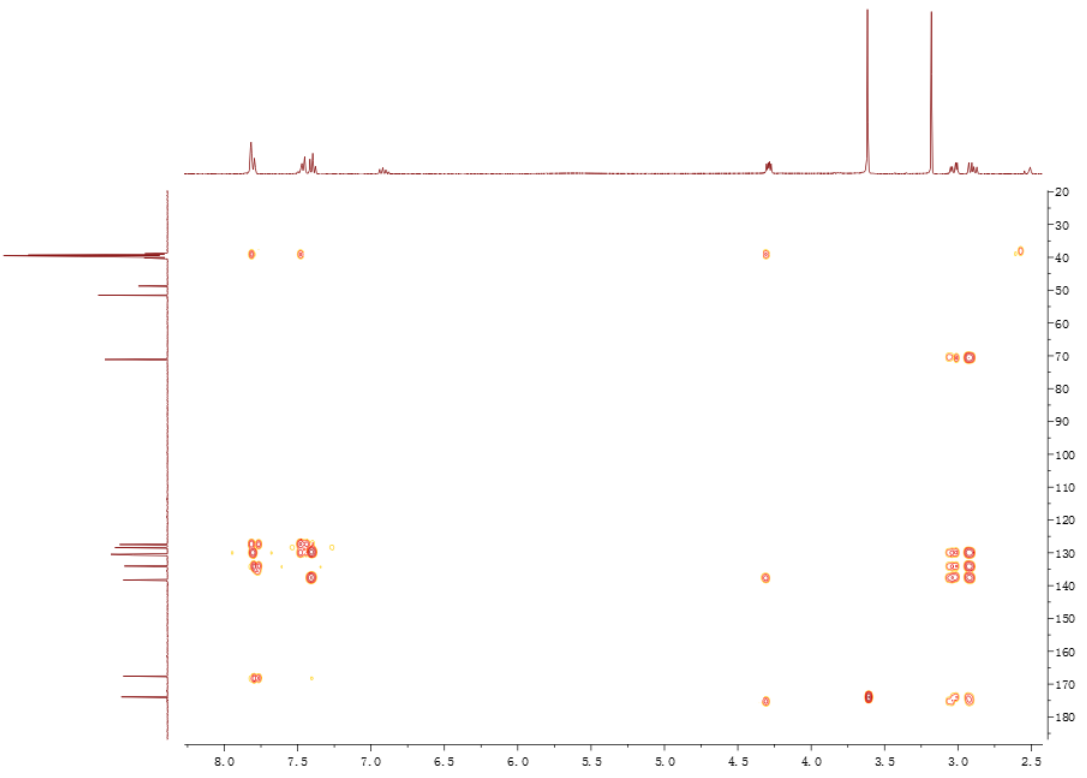

Figure S32. HMBC spectrum of plumeriapropionic E (**5**)

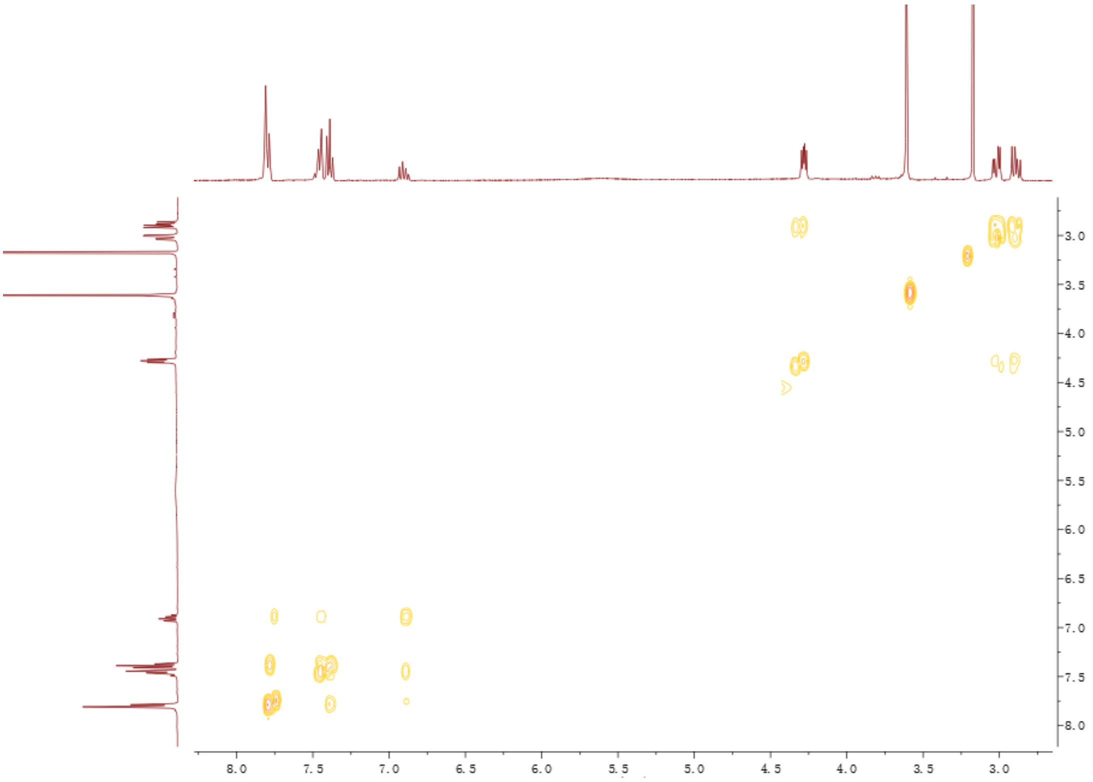

Figure S33. ^{1}H-^{1}H COSY spectrum of plumeriapropionic E (**5**)

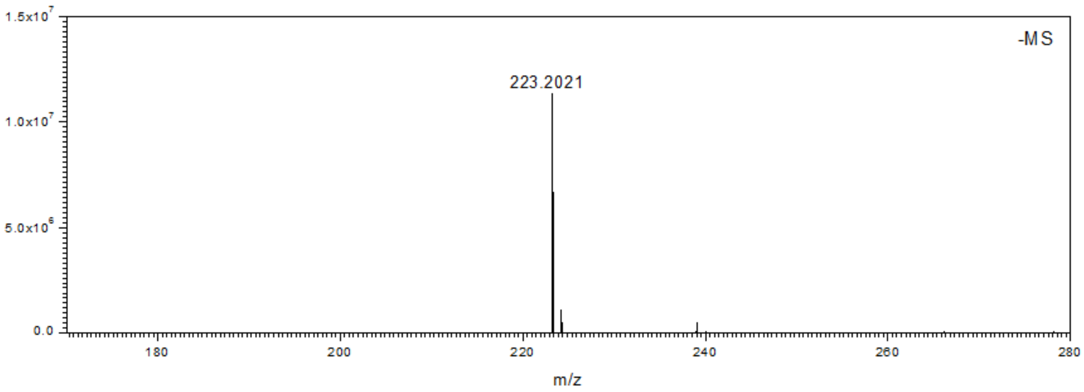

Figure S34. HRESIMS of plumeriapropionic E (**5**)

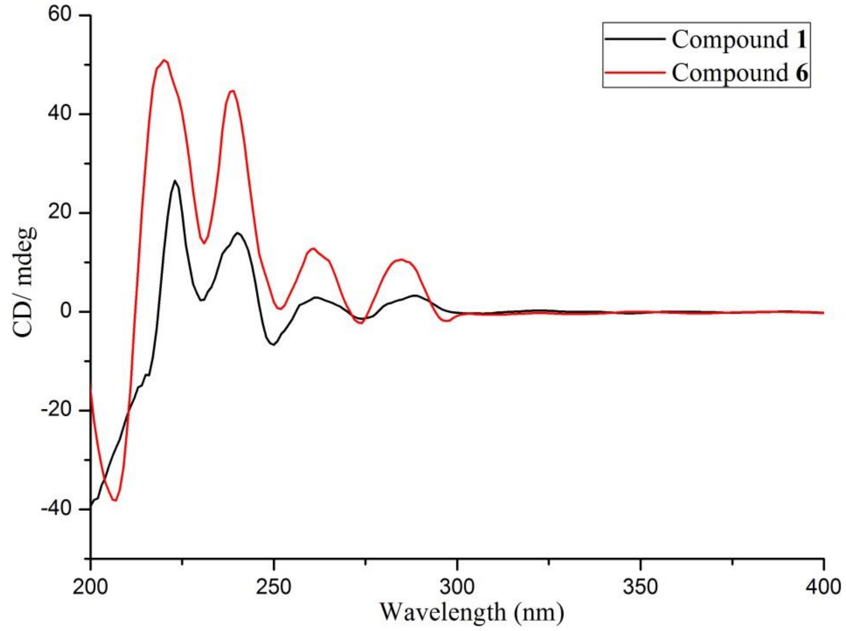

Figure S35. ECD spectra of **1** and **6**.

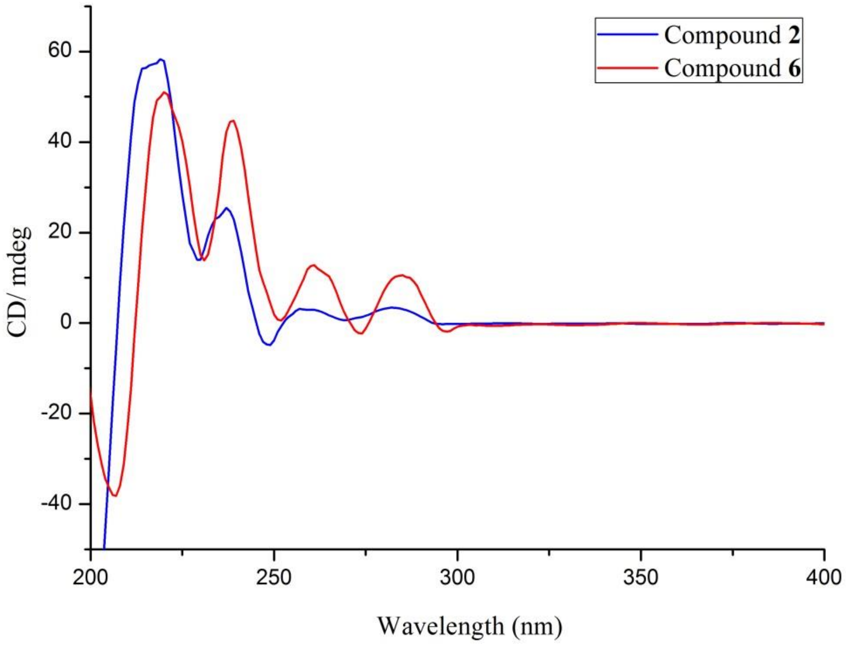

Figure S36. ECD spectra of **2** and **6**.

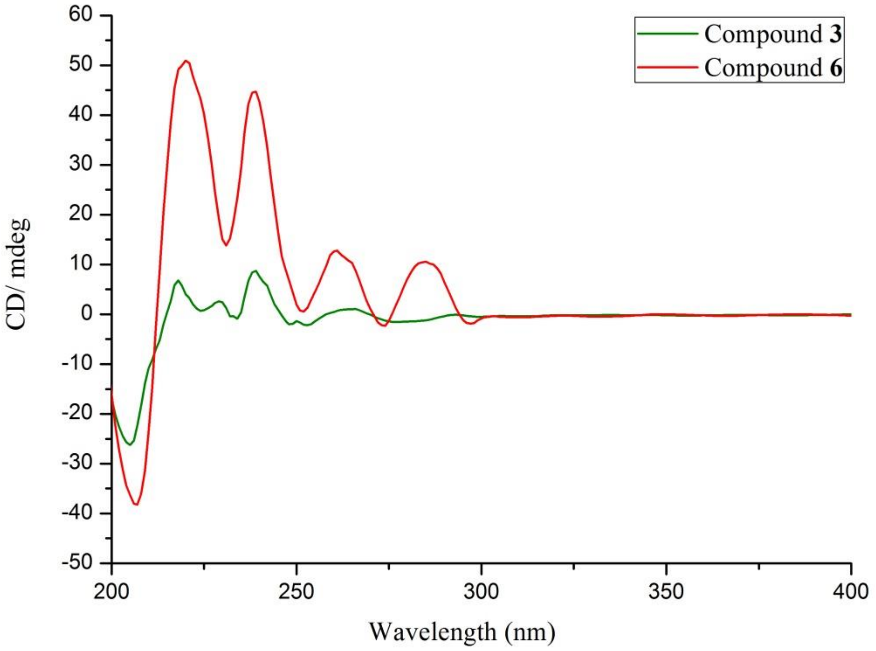

Figure S37. ECD spectra of **3** and **6**.

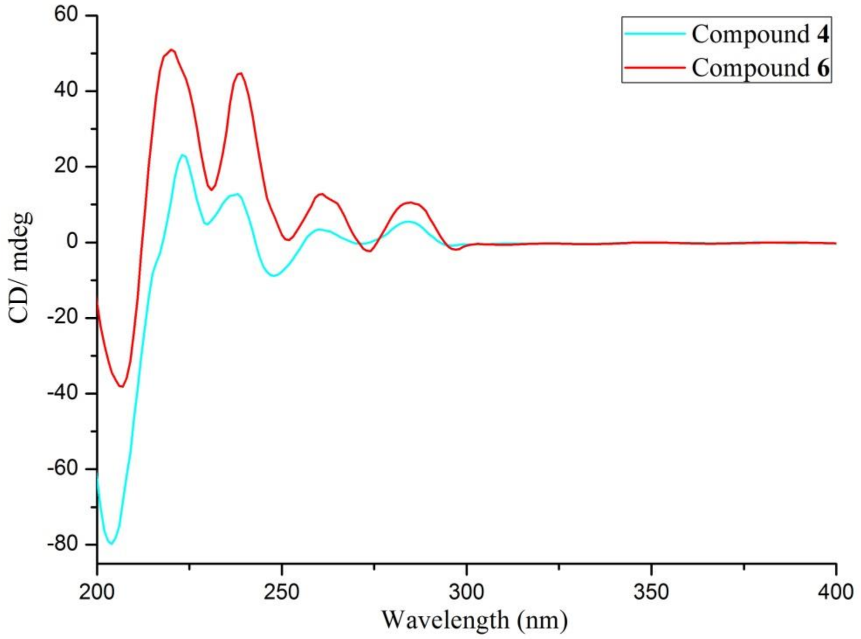

Figure S38. ECD spectra of **4** and **6**.

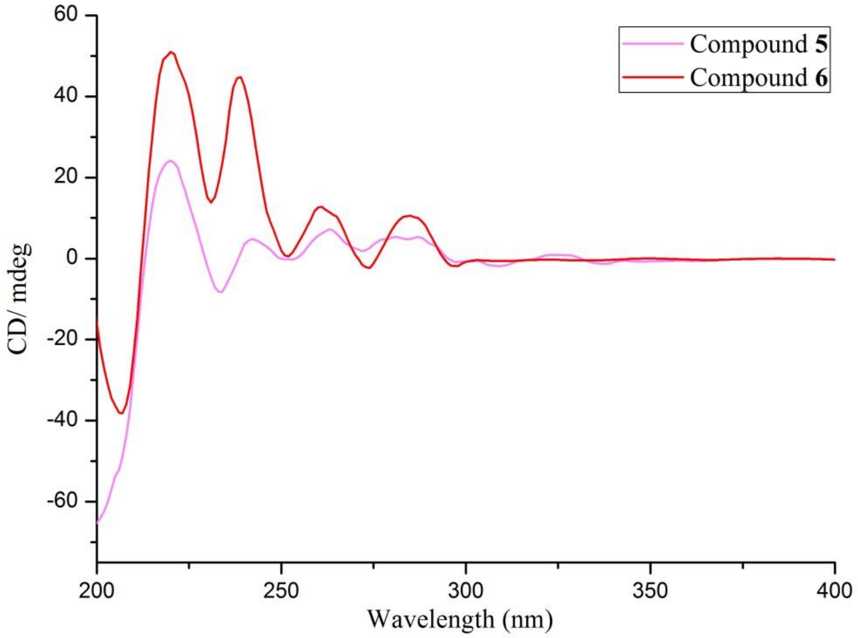

Figure S39. ECD spectra of **5** and **6**.
